# Preventing *E. coli* Biofilm Formation with Antimicrobial Peptide-Functionalized Surface Coatings: Recognizing the Dependence on the Bacterial Binding Mode Using Live-Cell Microscopy

**DOI:** 10.1021/acsami.3c16004

**Published:** 2024-01-31

**Authors:** Adam Hansson, Eskil André Karlsen, Wenche Stensen, John S. M. Svendsen, Mattias Berglin, Anders Lundgren

**Affiliations:** † Department of Chemistry and Molecular Biology, University of Gothenburg, Gothenburg 40530, Sweden; ‡ Amicoat A/S, Sykehusvegen 23, Tromsø 9019, Norway; § Department of Chemistry, UiT The Arctic University of Norway, Tromsø 9037, Norway; ∥ Department of Chemistry and Materials, RISE Research Institutes of Sweden, Borås 50115, Sweden; ⊥ Centre for Antibiotic Resistance Research (CARe), University of Gothenburg, Gothenburg 41346, Sweden

**Keywords:** antimicrobial peptides, antibiotics resistance, biofilms, fimbriae, surface coatings, live-cell microscopy, microfluidics, image analysis

## Abstract

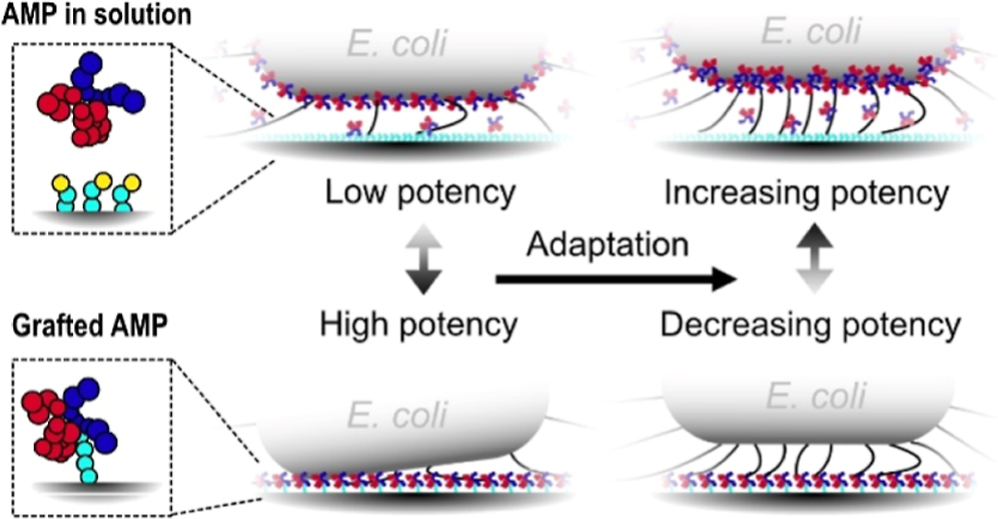

Antimicrobial peptides (AMPs) can kill bacteria by destabilizing their membranes, yet translating these molecules’ properties into a covalently attached antibacterial coating is challenging. Rational design efforts are obstructed by the fact that standard microbiology methods are ill-designed for the evaluation of coatings, disclosing few details about why grafted AMPs function or do not function. It is particularly difficult to distinguish the influence of the AMP’s molecular structure from other factors controlling the total exposure, including which type of bonds are formed between bacteria and the coating and how persistent these contacts are. Here, we combine label-free live-cell microscopy, microfluidics, and automated image analysis to study the response of surface-bound *Escherichia coli* challenged by the same small AMP either in solution or grafted to the surface through click chemistry. Initially after binding, the grafted AMPs inhibited bacterial growth more efficiently than did AMPs in solution. Yet, after 1 h, *E. coli* on the coated surfaces increased their expression of type-1 fimbriae, leading to a change in their binding mode, which diminished the coating’s impact. The wealth of information obtained from continuously monitoring the growth, shape, and movements of single bacterial cells allowed us to elucidate and quantify the different factors determining the antibacterial efficacy of the grafted AMPs. We expect this approach to aid the design of elaborate antibacterial material coatings working by specific and selective actions, not limited to contact-killing. This technology is needed to support health care and food production in the postantibiotic era.

## Introduction

The rise of antibiotic resistance in common pathogens is a looming threat to patients worldwide as it disarms our most potent tools against infectious diseases.^1^ In medical settings, the issue is exacerbated by bacteria’s tendency to colonize and form resilient biofilms on abiotic surfaces found in medical devices such as implants, catheters, and medical instruments.^2^ Biomaterial-associated infections with resistant bacteria already pose a heavy burden on the resources of medical clinics and result in bad health outcomes for the patients.^3−6^ We are particularly running out of treatments against Gram-negative bacteria like *Escherichia coli*; most pathogens on WHO’s priority list of antibiotic-resistant bacteria belong to this subgroup.^1^ One straightforward approach to escaping biofilm formation is to inhibit bacterial colonization by chemically attaching substances that suppress bacterial growth, or binding, to the interface of biomaterials. Resistance development is less likely to occur with grafted substances than with substances leaking to the environment around a device. Due to the covalent attachment, bacteria will experience a permanent, high concentration of antibacterial substance in the close vicinity of the surface acting selectively only on those bacteria that bind to the surface. In many applications, the main aim is to prevent the formation of resilient biofilms; thus, coatings that slow down the growth of bacteria, or the development of biofilm-promoting phenotypes specifically, without killing, may fulfill this purpose too. Finally, the choice of antibacterial substance(s) may include molecules that have a very broad mode of action rather than targeting a specific biochemical mechanism. Although resistance development toward such substances remains possible, it would imply so large changes of the bacteria that it is ecologically disadvantageous in the long term.

Antimicrobial peptides (AMPs) are an archetype of the molecules with potential to replace classical antibiotics in this type of application.^7−9^ These are amphiphilic peptides that can insert into the cell membrane(s) of bacteria and disturb their membrane homeostasis,^10^ eventually leading to cell death. Insertion is facilitated by a high content of cationic amino acids, leading to selective binding of AMPs to the bacterial membrane, which have more anionic lipids than other cell membranes.^11^ Most studied AMPs have a natural origin as being part of the innate defense of different prokaryotic or eukaryotic species.^11,12^ The use of natural AMPs for antibacterial products has however been marginally successful; their length and the dependence on native sequence for their function makes them sensitive to environmental settings and to enzymatic digestion, prone to cause off-target cytotoxicity and lead to high production cost.^8^ Through studies of the structure–activity relationship for natural AMPs, novel peptides that are smaller, cheaper, and more stable have been designed.^13−15^ The smallest pharmacophore was found to have only three residues^16^ and has proven to be a good candidate for usage in AMP-based therapies and products against common bacterial and fungal pathogens.^17−20^

Since AMPs lack an apparent secondary structure, the antibacterial mechanisms of short AMPs remain elusive but is more likely due to interference with membrane protein function than formation of pores.^21−23^ Still, the efficacy and susceptibility profiles of short AMPs are very sensitive to minor alteration of the order and frequency at which amino acids appear^13,24,25^ to peptide cyclization^26^ and to the position and stereochemistry of synthetic side-chains added to the pharmacophore.^27^ Given the wealth of possible combinations, exploring which molecular configuration is best for a certain application presents a real challenge. For AMP-based coatings, the situation is further exacerbated since the efficacy of an antibacterial surface does not necessarily correlate with the intrinsic potential of the AMPs in solution, as measured by the classical minimum inhibition concentration (MIC) value.^28,29^ In solution, all the molecule’s rotational angles are unrestricted, and thus, an AMP can bind to the bacterial membrane in the most favorable configuration given by the electrostatic interaction. When AMPs are covalently attached, the molecular dynamics will be restricted and the AMP exposure will rely on the small geometrical and mechanical details of the bacteria–surface interactions and their persistence. The potency of tethered AMPs will, for example, increase if the cationic amino acids are positioned closer to the anchoring position than the hydrophobic since this promotes the directed insertion into the membrane.^28^ Furthermore, there are methodological aspects that can explain the difference too: While MIC is a measure of proliferation under conditions supporting bacterial growth, i.e., in rich medium under agitation, the potency of tethered AMPs is commonly measured as a reduced, normalized viability under conditions that poorly support surface growth, e.g., in stagnant buffer or heavily diluted growth media.^30^ The liquid environment can have a direct effect on the magnitude of the interaction between an AMP and the bacterial membrane,^31−33^ and nutrient deficiency may impede the bacteria from responding to the surface binding by the development of biofilm-specific phenotypes and activation of stress responses that serve to maintain membrane homeostasis.^34−38^

It should indeed be recognized that in any clinical application, the antibacterial efficacy of a coating relates to a combination of its ability to reduce surface growth, to prevent binding, and to inhibit development of biofilm-specific phenotypes, among which the last are often heterogeneously distributed among bacteria in a population.^39,40^ Specialized methods to analyze the antibacterial efficacy of coatings, like the CERTIKA test, which aim to measure the release of bacterial cells from a biofilm established on the test surface,^41^ and the popular live/dead staining procedure are usually implemented as artificial end-point measurements that do not monitor the biofilm formation dynamically with single-cell resolution. It is therefore difficult to gain mechanistic insights from these tests, and since the procedures require manual intervention, the results are also sensitive to the handling of washing and staining.^42−44^ In contrast, mechanistic understanding of the bacteria’s response to AMP has been made available from time-resolved live microscopy of single cells featuring fluorescent probes that continuously indicate membrane permeability, oxidative status, etc.^21,45−47^ The corresponding procedures and analysis are, however, more complex, limiting the settings under which such tests can be done. Thus, there is an imminent need for medium-throughput methods that can monitor dynamically the early events of biofilm formation on antibacterial coatings under conditions that closely resemble the end-usage, therethrough delivering quantitative information about all their different modes of actions.

Herein, we have built a microfluidic-based platform for live cell microscopy and subsequent automated image analysis using the simplest possible microscopy without any labels or probes. As proof-of-concept, we synthesized an azide-conjugated version of the potent tripeptide AMP AMC-109,^17,48^ which was tethered to the floor of the microfluidic channel using copper-catalyzed alkyne–azide cycloaddition (click reaction),^49^ and analyzed the binding to and growth of individual *E. coli* bacteria on this surface. To distinguish effects specific to details of the tethering from those specific to the AMP, bacteria were also bound to natural, mannose-modified surfaces and charged with the original pharmacophore and its azide-modified counterpart in solution. This approach allowed us to distinguish how *E. coli*’s adaptation to biofilm life form interferes with the efficacy of the AMP coating: While initially the grafted AMPs inhibited the growth of bound bacteria more efficiently than did soluble AMPs, *E. coli* eventually increased their expression of type-1 fimbriae, leading to a change in their binding mode, which completely diminished the coating’s impact. The presented method provides a more holistic view of the coating’s antibacterial potential than other methods. It will therefore be useful to guide the development of antibacterial coatings, particularly for applications with specific and/or selective antibiofilm actions beyond the current paradigm of contact killing surfaces.^50^

## Materials and Methods

### Synthesis of the AMP AMC-25-04

Amino-azido derivate of PEG200 was prepared as described by Jiang et al.^51^ and reacted with diglycolic anhydride to provide the azido-PEG-COOH linker. 1 equivalent (equiv) azido-PEG-COOH was coupled to 1 equiv AMC-109^52^ (Amicoat AS, Norway) using 1.2 equiv HBTU and 4.8 equiv TEA to gain AMC-25-04. The crude peptide was purified by preparative high-performance liquid chromatography (HPLC) and lyophilized to yield TFA-salt. The purity was >95%, as determined by HPLC. The product was confirmed with NMR (Figure S1) and ESI-MS (MH^+^, monodisperse, calculated for C_55_H_90_N_15_O_9_^+^: 1104.7040, observed 1104.7009).

### Microfluidic Channel Assembly and Surface Modifications

Microfluidic channels were prepared by mounting a square glass capillary with inner diameter 0.8 × 0.8 mm^2^ (VitroCom, USA) on a microscopy slide using UV-curable adhesive (NOA 68, Norland Products inc., USA). Pipette tips (Gel-loading Pipette Round Tips, VWR) were inserted into and fixated with glue at both ends of the capillary. The interior of the channels, as well as the coverslip glass surfaces used for fluorescence microscopy and time-of-flight secondary ion mass spectrometry (ToF-SIMS) analysis, was cleaned by immersion overnight with Hellmanex III cleaning solution (2%, Hellma GmbH, Germany) and rinsed with water (Milli-Q, Merck Life Science), followed by immersion in sulfuric acid (2 M, Sigma-Aldrich 99.9%) for 1 h, followed by extensive rinsing with water. The cleaned surfaces were rinsed with ethanol (99.5%, Solveco, Sweden) and then silanized by immersion in 10% solution of *O*-(propargyloxy)-*N*-(triethoxysilylpropyl)urethane (90%, ABCR, Germany) in ethanol (99.5%, Solveco, Sweden) for 1 h. The silanized capillaries and coverslips were rinsed with ethanol, followed by water, and modified with AMP AMC-25-04 or α-mannose-PEG3-azide (>95%, Sigma-Aldrich) or azide-fluor 488 (>90%, Sigma-Aldrich), using copper-catalyzed alkyne-azide cycloaddition (CuAAc, click-chemistry).^49^ This was followed by immersion of the surfaces for 10 min in a click reaction solution containing 33 μM azidated reactant, 17 mM aminoguanidine hydrochloride (Sigma-Aldrich), 75 μM CuSO_4_ (Sigma-Aldrich), 250 μM tris(3-hydroxypropyltriazolylmethyl)amine (THPTA, Tokyo Chemical Industry Co., Ltd.), and 500 μM ascorbic acid (Merk) diluted in PBS buffer (pH 7.4), whereupon the modified surfaces were rinsed with water. After finishing the experiments, the pristine glass surface of the channels was regenerated through extensive cleaning with Hellmanex III overnight followed by immersion in sulfuric acid (2 M, Sigma-Aldrich 99.9%).

### Verification of Surface Modifications

The glass surface modifications were verified by contact angle measurements with fluorescence microscopy and ToF-SIMS. Contact angle measurements of three silica wafers (10 mm × 10 mm) coated with either silane or AMC-25-04, respectively, were acquired using a DSA100 instrument from Krüss GmbH (Hamburg, Germany). A 5 μL water droplet was deposited onto the wafer and imaged after a 10 sec delay. The images were analyzed using ImageJ version 1.53t. Coverslip glass modified with Azide-fluor 488 by click reaction, with and without added CuSO_4_ (see above), was gently scratched with a needle to provide some stripes on the surface devoid of fluorophores to enhance contrast in the image. The different surfaces were then imaged while being immersed in water using an upright fluorescence microscope (Axioskop 20, Carl Zeiss Microscopy) equipped with a water-immersion objective (W plan-apochromat 63*x*/1.0, Carl Zeiss Microscopy) using the same exposure time. ToF-SIMS (TOFSIMS IV, IONToF GmbH, Germany) was used to analyze the presence and spatial distribution of peptide fragments before and after their surface attachment to silanized glass. The instrument was operated using 25 keV Bi^3+^ primary ions at a pulsed current of 0.1 pA (cycle time 150 μs, width 1.2 ns). The samples were analyzed in the bunched mode at an analysis area of 500 × 500 μm^2^ (resolution of 256 pixel). The average of 25 scans at an acquisition time of 100 sec was used when acquiring data using SurfaceLab version 6.7 software from IONToF GmbH (Münster, Germany).

### Bacteria and Growth Media

Versions of K12 *E. coli* wild-type strain MG1655 were used throughout all experiments. The wild-type strain (referred to as WT *E. coli* in the text) used in most of the experiments was provided green fluorescence (GFP) and resistance to Kanamycin by transformation with plasmid pBE1-mGPFmut2.^53^ A strain that overexpresses type-1 fimbria (referred to as *E. coli*-*Fim*+ in the text) was made by transformation of WT *E. coli* with plasmid pPKL91, which promoted fimbriae expression by increasing the intracellular concentration of the regulating protein FimB.^54^ A strain that lacks type-1 fimbriae (referred to as *E. coli*-Δ*FimA* in the text) was made by deletion of the *fimA* gene encoding the major structural protein (FimA) of the fimbria from the chromosome of *E. coli* MG1655. For this, Δ*fimA::kan* was transduced from the KEIO collection strain JW4277^55^ by standard methods to MG1655, selected for Kanamycin resistance, and the locus was verified by PCR. The fimbriated and nonfimbriated phenotypes were confirmed by yeast agglutination. The bacteria were kept in deep-frozen glycerol stocks and on a weekly basis plated and grown on LB-Agar supplemented with the appropriate antibiotics. For live-cell microscopy experiments, a single colony was selected from a plate, inoculated into LB media supplemented with 50 μg/mL Kanamycin and grown overnight at 37 °C. The overnight cultures were gently centrifuged to remove aggregated bacteria. The supernatant retrieved after centrifugation, which typically had OD_600_ ≈ 0.1, was diluted 1:1 in fresh LB media supplemented with Kanamycin and grown at 37 °C until 0.35< OD_600_ < 0.6.

### Live-Cell Microscopy

The microfluidic channel was mounted under a microscope (Axioskop 20, Carl Zeiss Microscopy) equipped with a stage heated to 37° (SKE, Italy). The channel was connected to a syringe pump (NE-300, New Era Pump Systems) via polypropylene tubing. A pipette tip attached to the tubing by UV-curable adhesive (NOA 68, Norland Products inc., USA) worked as an adapter between the tubing and the pipette tip attached to the channel. The lower surface of the channel was imaged using a water-immersion objective (W plan-apochromat 63*x*/1.0, Carl Zeiss Microscopy), and footage was acquired using a microscope camera (Axiocam 305 Color, Carl Zeiss Microscopy) at 2 fps. Bacteria grown to the log-phase in LB media (see above) were taken directly from the incubator, transferred to a syringe, and injected into the channel, first for 10 min at a flow rate of 100 μL/min to equilibrate the system and then for another 10 min at a lower flow rate of 20 μL/min to promote bacterial binding. The first syringe was then replaced with a new syringe containing LB media or LB media supplemented with 100 μM AMPs, which was injected at a flow rate of 100 μL/min for approximately 3 h. Notably, with the present channel dimensions, the flow rate of 100 μL/min translates into a flow speed of approximately 30 μm/s at 1 μm separation from the surface where the bacteria sit^56^ (Figure S2). At a lower flow speed, *E. coli* grows significantly slower and does not form microcolonies well (data not shown). Therefore, if channels with other dimensions are used, it is important to adapt the flow rate accordingly.

### Image Analysis and Segmentation

Image analysis of footage was done using a script written in MATLAB (MATLAB Version: 9.13.0.2049777 [R2022b], The MathWorks Inc., USA) featuring functions of the Image Processing Toolbox. For the analysis of bacterial length and growth rate (GR), nonbound bacteria were excluded from analysis by averaging every 20 consecutive frames, reducing the effective frame rate from 2 to 0.1 fps. For the analysis of bacteria’s small motions around their major axis, footage was analyzed at the original frame rate for periods of 2 min. All images were first corrected for uneven illumination and the background was subtracted. The resulting image was used to construct a mask that excluded all nonbacterial objects by, in the following order: subtraction of a user-set constant intensity value, two rounds of median filtering, marker-controlled watershed segmentation, and application of shape/size criteria. Using this mask, properties of the individual bacteria were extracted from the original frame; the length was measured as the major axis of the best-fitting centroid. Bacteria in subsequent frames were stitched together to trajectories if their footprints overlapped and their length changed <25%. To handle issues with bacteria temporarily “disappearing” from the segmented mask, e.g., due to focus drift, a bacterium remained a member of the same trajectory although it was not visible for some short time (up to 1 min) if it reappeared with unaltered appearance. Else, new trajectories started if bacteria divided or appeared at new positions.

### Analysis of Growth Rate and Mean Binding Time from Growth Trajectories

The evolutions of population GR, GR(*t*), cell length, *L*(*t*), and the overall mean binding time, *T*_1/2_, were extracted from trajectories of growing cells using MATLAB (MATLAB Version: 9.13.0.2049777 [R2022b], The MathWorks Inc., USA) featuring functions of the Curve Fitting Toolbox and the Statistics and Machine Learning Toolbox. The momentaneous GR of a bacterium was determined by the best linear fit to cell length data extending ±5 min around each time point of a trajectory. Trajectories shorter than 10 min and the first and last 5 min of each trajectory (which are prone to noise) were thus not included. The experimental time axis was divided into 20 min intervals, and the mean GR for each bacterium within each time interval was calculated. The distribution of mean GRs and lengths across the population within each time interval is shown in the boxplots. The time evolutions of the median GR, GR(*t*), and length, *L*(*t*) for a population were estimated by fitting Gompertz functions to the data of the boxplots. The duplication time, *T*_2_, was estimated from these functional dependencies assuming that *T*_2_ (*t*) = (2/3) × (*L*(*t*_end_)/GR(*t*)). The number of bacteria on the surface at any time after binding, *N*(*t*), will depend both on *T*_2_(*t*) and on the rate of bacteria release from the surface, indicated by the mean binding time, *T*_1/2_, through the relation
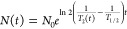
1where *N*_0_ is the number of bacteria at the surface right before bacteria starts to divide, which was approximated by the time of the inflection point of the Gompertz fit. *T*_1/2_ was determined from the best fit of eq 1 to plots of the number of bacteria versus time.

### Measurement of Growth Curves in Small Batch Cultures

Growth curves for *E. coli* in bulk subjected to different concentrations of AMPs AMC-109 and AMC-25-04, respectively, were acquired using a plate reader (POLARstar OMEGA, BMG Labtech, Germany). Solutions of *E. coli* grown to log-phase were prepared as described above for the live-cell experiments but diluted just before use to OD 0.10 in LB media preheated to 37°. Working in a cabinet heated to 37 °C and using only preheated solutions and plates, 150 μL of the bacteria solution was loaded to the wells of a 96-well plate (TTP, Techno Plastic Products AG, Switzerland). Another 150 μL of the same bacteria solution also containing 200 μM AMP was then added to the first row of the plate, thereby diluting the AMP concentration to 100 μM. This concentration was then serially diluted by factor 2 in several steps by transferring 150 μL of the bacteria-AMP solution downward along the columns of the plate from the first row to the second row and so on. Since the AMPs form particles that increase the OD of the solutions in a concentration-dependent manner, controls were made by diluting LB media containing only AMPs but no bacteria in the same way. The different AMPs and bacteria AMP-solutions were loaded in triplicate on the same plate, i.e., three columns per solution. The plate was put in the plate reader, which was operated at 37 °C, and the OD at 600 nm was measured in all wells every 20 min for 6 h. The presented growth data are the mean OD for the bacteria-AMP solution subtracted by the mean OD of the AMP solution with the same concentration.

## Results and Discussion

### Coating of Microfluidic Channels with AMPs through Click Reaction

**Figure 1:**
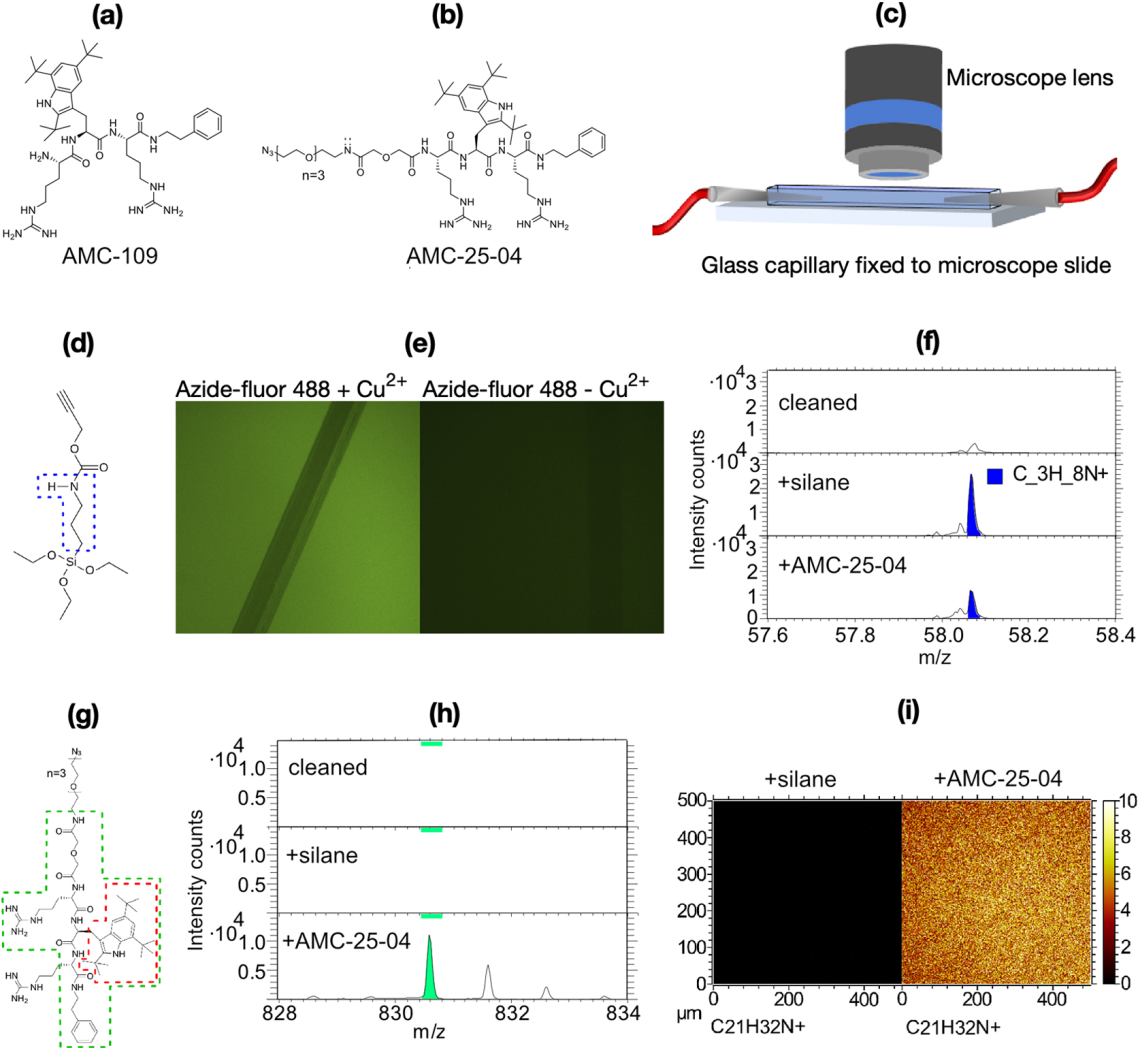
Surface modification of microfluidic channels. (a) Chemical structure of AMC-109. (b) Chemical structure of AMC-25-04. (c) Cartoon illustrating how the microfluidic channel was set up. (d) Chemical structure of *O*-(propargyloxy)-*N*-(triethoxysilylpropyl)urethane. The blue broken line indicates the potential origin of fragment C_3_H_8_N^+^. (e) Fluorescence micrographs of silane-modified glass showing a glass surface after modification with Azide-fluor 488 in the presence of all reagents needed for click reaction (left panel) and a negative control where copper ions were omitted (right panel). The surfaces were gently scratched with a steel needle. (f) Part of the ToF-SIMS spectrum showing total intensity counts of a clean glass surface (top), a surface modified with silane (center), and a surface modified first with silane and then with AMC-25-04 through click reaction (bottom). The blue-shaded peak corresponds to the ionized fragment C_3_H_8_N^+^ (*m*/*z* ≈ 58.07, cf. panel [d]). (g) Chemical structure of AMC-25-04 where the ionized fragment with *m*/*z* ≈ 830.6 is indicated by a green broken line and the fragment C_21_H_32_N^+^ (*m*/*z* ≈ 298.8) corresponding to tri-*tert*-butyl tryptophan is indicated by a red broken line. (h) Part of the ToF-SIMS spectrum analogous to that of panel (f) highlighting the green-shaded peak corresponding to the fragment with *m*/*z* ≈ 830.6 from the AMC-25-04 molecule. (i) ToF-SIMS micrographs showing the distribution of the ionized fragment C_21_H_32_N^+^ over an area of 0.5 × 0.5 mm^2^ (256 × 256 pixels) of surfaces modified with only silane (left) and silane + AMC-25-04 (right). The color scale shows the number of fragments detected for each pixel.

We aim to develop a method that can be used to rank the antibiofilm potential of different coatings and simultaneously provide some mechanistic information about the interaction between bacteria and the AMP-modified surface. This includes the bacteria’s biological response to binding, in particular changes of the binding mode, which are not detectable with conventionally employed methods. To isolate this aspect of the coating’s efficacy, we use only one type of AMP, either dissolved in the growth media or covalently attached, and one tethering strategy in this initial proof-of-concept study. This because the amino acid sequence variation, the tether chemistry, and the tether attachment point on the AMP are known to strongly influence the activity of the grafted AMPs.^28,29,57−60^ The minimalistic approach is suitable considering the scope of the study; it should however be emphasized that the design may not be optimal for other applications. As basis for the coating, we chose to use AMC-109 (also known as LTX-109), which is a synthetic tripeptide AMP that has proven efficient against common bacterial and fungal pathogens.^17−20^ Its primary structure corresponds to R–W–R, giving the peptide a net charge of +3. The central tryptophan is modified with three bulky *tert*-butyl groups, and the C-terminal is capped with an ethylphenyl group, which enhances the overall hydrophobicity of the peptide and protects it against enzymatic digestion^52,61^ (Figure 1a).

To make the AMP surface coating of this pharmacophore, we created the peptide AMC-25-04 by attaching an azide-terminated poly(ethylene glycol) linker to AMC-109 (Figure 1b). Generally the addition of the azide group makes it possible to conjugate an AMP to a surface or matrix displaying alkyne functionality by copper-catalyzed alkyne–azide cycloaddition, commonly referred to as “click-chemistry”.^49^ The azide can be positioned differently along the peptide chain for example by replacing the amine of a lysine side chain with an azide and potentially be tethered through a PEG linker, which could be of various lengths. In a recent study, a series of pentapeptide AMPs were conjugated this way to gold surfaces modified with self-assembled monolayers of alkyne-terminated thiols.^29^ This study and previous studies featuring other conjugation methods highlighted structure–activity relations specific for surface-grafted short AMPs. Most importantly, cationic and hydrophobic amino acids should be positioned relative to the surface linker in a way so that AMP insertion into the membrane is facilitated.^28^ Native AMPs may become more potent if presented on tethers of certain length or chemical composition since these factors can aid the AMPs in attaining their biologically active conformation.^57,58^ Less is known about how the nature of the tether influences the performance of small optimized AMPs. When such peptides were immobilized on, or together with, high-molecular-weight hydrophilic polymers, the resulting coatings showed very good antimicrobial performance. The effect could however mainly be attributed to the antifouling properties of the polymers.^59,60^ We wanted to study details of bacterial adhesion, which would be easier if bacteria bind readily to the coated surface. The peptide AMC-25-04 was therefore made by attaching a short PEG linker (DP = 3) to the *N*-terminus of AMC-109. We foresee that this tether will help to direct the AMP so that the interaction between its hydrophobic components and the underlying surface is prevented, yet it will not interfere with bacterial adhesion. Notably, the modification also reduces the net charge of the peptide to +2. The final structure was confirmed by NMR (Figure S1) and mass spectrometry, and the product was purified to >95% using HPLC.

In this work, we expand previous approaches to measure the efficacy of AMP coatings by monitoring in real time bacterial binding and growth on it in a microfluidic channel (Figure 1c). The use of microfluidics is optimal for live-microscopy experiments since it allows the environmental conditions to be tuned to resemble natural conditions. Using microfluidics also removes inherent problems associated with batch-cultures such as the inoculum effect^62^ and variability caused by manual interference (e.g., rinsing and staining steps) during the experiment. This increases reproducibility and accordingly allows us to also measure small differences between samples. Several applications have already exploited the advantages of microfluidics for testing the susceptibility of bacteria growing in biofilms to antibiotics provided in solution.^63−66^ To make a flexible microfluidic platform that works for the evaluation of antibacterial coatings, we first modified the inner walls of the glass capillary with silane presenting an alkyne functional group (Figure 1d), allowing further conjugation via generic click chemistry. Fluorescence microscopy showed that azide-conjugated fluorophores readily bound to silane-modified glass surfaces in the presence of all reagents needed for click reaction but very sparsely if copper ions were omitted (Figure 1e), confirming the reactivity of the silane modification.

For the control experiments, i.e., in experiments without AMPs and where AMPs were provided in the growth media but not as a coating, d-mannose was conjugated to the alkynes on the silanized glass surfaces. Most *E. coli* strains bind readily to d-mannose-coated surfaces via their type-1 fimbriae.^67^ The AMP-coated surfaces were made similarly by the attachment of AMC-25-04. This increased the hydrophobicity of the surfaces, and the water contact angle increased from 38 ± 2° to 47 ± 1°, confirming that the hydrophobic side chains of AMC-25-04 rather than the hydrophilic PEG tethers are exposed. To further verify the presence and homogeneity of the AMP coating, we used ToF-SIMS analysis (Figure 1f–i). Glass surfaces modified with only silane showed a high flux of an ionized molecular fragment C_3_H_8_N^+^ (Figure 1f). This corresponds to the central part of the silane (Figure 1d). Upon binding of AMC-25-04, the flux decreased relative to the surface with only silane, which is expected due to shielding. Note that the peak visible in the spectrum for the clean surface corresponds to another, slightly heavier, fragment. Several unique fragments with high *m*/*z* values appeared after AMP binding. Figure 1h shows a spectrum highlighting an ionized fragment encompassing the full AMC-25-04 but for the PEG-linker and one of the arginine side chains (Figure 1g). The fragment C_21_H_32_N^+^ corresponding to the artificial amino acid tri-*tert*-butyl tryptophan (cf. Figure 1g) was found to coat the surface homogeneously on the length scale corresponding to that observable with optical microscopy (Figure 1i).

### Bacterial Binding and Growth on Mannose-Coated Surfaces under Favorable Conditions

**Figure 2:**
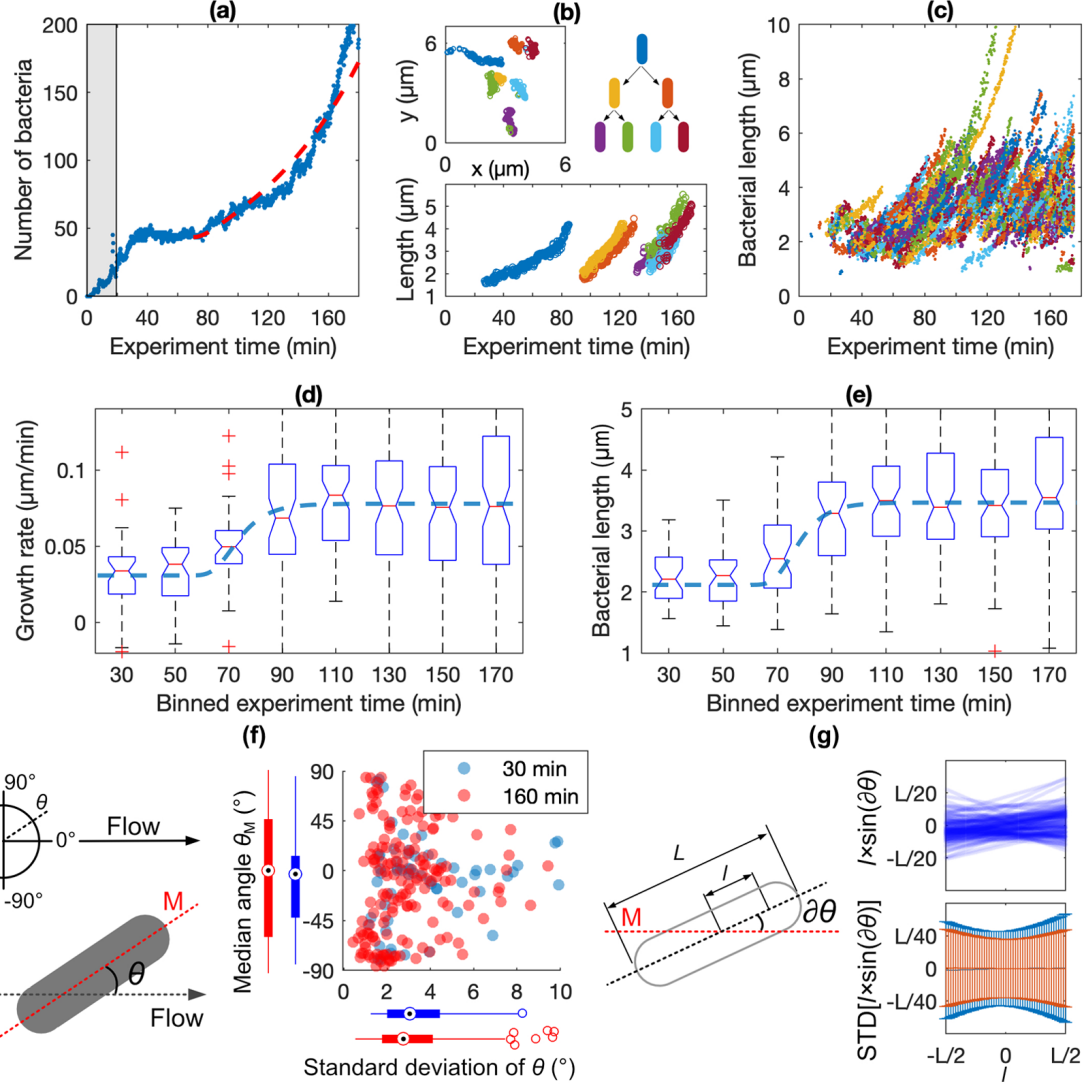
Method for the analysis of binding and growth. (a) Number of identified bacteria on the imaged surface from the beginning to the end of the experiment. The gray-shaded region indicates the period of bacteria injection. The red broken line indicates the best fit of eq 1 to the number data. (b) Selection of traces showing the formation of a microcolony plotted in the spatial (upper panel) and time (lower panel) domains, respectively. The mother–daughter relations are shown by a color code. (c) Plot showing all data points, color-coded according to the tracing procedure for which GRs were calculated. (d) Boxplot showing the distribution of mean GRs for the bacteria present divided into 20 min intervals. (e) Boxplot showing the distribution of the mean lengths of the bacteria for which GR was presented in (d). (f) Cartoon diagram and plot detailing the analysis of bacterial alignment, i.e., the angle *θ* between a bacterium’s major axis and the flow direction. The combined scatter and boxplot show the median angle, *θ*_M_, versus the standard deviation (STD) of *θ* for each bacterium at an early (30 min, blue points) and a late (160 min, red points) time point of the experiment. (g) Cartoon diagram and plots detailing the analysis of bacteria’s wiggling movements around their median major axes. The scatter plot in the upper panel shows for a single example bacterium the instantaneous separations, *l* × sin(∂*θ*), between each position *l* along the bacterium’s major axis with length *L* and the median major axis *M*. The bar plot in the lower panel shows the distribution (standard deviations) of the instantaneous separations, *l* × sin(∂*θ*), for all positions *l* of all bacteria present early (30 min, blue bars) and late (160 min, red bars) in the experiment.

In all live-cell microscopy experiments, the bacteria were dispersed in growth media and injected into the microfluidic channels for 20 min at a low flow rate, which facilitates their binding to the channel bottom. In the positive control experiments, the bacteria solution was then replaced with pure growth media and the flow was increased to get a sufficient flux of nutrients to support fast growth of bound bacteria (cf. Materials and Methods section and Figure S2). The binding and subsequent growth were filmed for >3 h at a rate of 2 fps with a microscope operated in brightfield mode. Figure 2 summarizes the results of the analysis applied to one of these control experiments.

First, automated image segmentation was implemented using an in-house developed MATLAB program; the bacterial objects were distinguished from other objects by their shape and size. The resulting plot of the number of bacteria versus time displays three phases: In the first phase (<30 min), the number of bacteria increases due to binding, in the second phase (30–70 min), the number remains almost constant, and in the last phase (>70 min), the number increases exponentially (Figure 2a). In a second step, trajectories showing the position in space and time of individual bacteria were constructed by stitching together the objects in subsequent frames. A new trace started if a bacterium appeared at a position not occupied in previous frames or when the cell divided (Figure 2b). Knowing a bacterium’s identity, the evolution of its characteristics can be analyzed straightforwardly. We chose to measure the bacterial length as our main characteristic since the length and its derivate with respect to time, i.e., the GR, are good indicators of the metabolism of a rod-shaped bacterium and whether it is subject to stress.^68−70^ Furthermore, for rod-shaped bacteria, the length measurement is relatively insensitive to noise or focus drift. Figure 2c shows the evolution of bacterial length for the individual bacteria (color-coded), and the boxplots of Figure 2e,f show the distribution of the GR and length, respectively, across the population at different times of the experiment. As detailed in the Materials and Methods section, only traces >10 min were used to construct these plots.

The data of Figure 2a–e, in combination, enable a deeper analysis of the early steps of biofilm formation. The first 40 min after binding, the bacteria displayed synchronized behavior characterized by a small size and low GR (Figure 2d,e). Only a few divisional events took place during this period (Figure 2c), which explains the plateau phase observed in the plot of the number of bacteria vs time (Figure 2a). Notably, the fact that the GR after binding is less than half of that typically observed for *E. coli* growing in a rich medium like LB does not per se indicate that the mannose-coated surface is “toxic” for the bacteria. Mannose is a natural in vivo receptor for fimbriated *E. coli* potentiating their binding to glycoproteins present, e.g., on the tissue of guts^71^ and the urinary tract.^72^ A mannose-coated surface is thus a good positive control since no adverse effects are expected due to the binding to this surface, and consequently, the bacteria may grow at their maximum rate under the given circumstances. Instead, the low GR is a well-known natural response to surface binding whereupon bacteria prioritize downregulation of the treats related to planktonic life and upregulation of those important for biofilm formation.^38^ After about 40 min, the GR accelerated and eventually saturated at about twice its initial value (Figure 2d). The cell length simultaneously increased and saturated at about 1.5 times its initial value (Figure 2e). This increase marks the end of the adaptation lag-phase and start of the biofilm growth phase as bacteria also begin to divide (Figure 2c). The final characteristic division time can be calculated from the GR and bacterial length (cf. Materials and Methods section) to be about 30 min, which is comparable to the characteristic division time in bulk LB media. Detailed inspection of the length traces for individual bacteria (Figure 2b,c) shows that cells grow linearly in the beginning of a cell cycle and faster, almost exponentially, when approaching division. This finding, which is in line with previous observations,^73^ explain the broadening seen for the distribution of GRs and cell lengths during the growth phase.

The growth of the biofilm depends not only on the rate at which cells divide but also on the rate at which cells leave the surface, a property related to the binding strength. To a first approximation, the characteristic binding time can be determined by fitting an exponential function to the part of the plot of the number of bacteria versus time that describe an exponentially increasing trend (Figure 2a, red broken line). The time constant obtained for the fitted curve is a function of both the division rate and the unbinding time. Since the former property is known, the latter can be determined to approximately 60 min for binding to the mannose-coated surface (cf. Materials and Methods section). We noted that this approach typically overestimates the binding strength in the beginning of the growth phase and underestimates it toward the later stage, indicating that this property changes, on the population level, with time. Indeed, it could be observed that after initial binding, the bacteria remained slightly mobile, moving in the direction of the flow, but eventually, the binding strength increased (Figure 2b, upper panel). This observation motivated us to also analyze the surface adhesion mode of the individual bacteria at the beginning (30 min) and at the end (160 min) of the experiment (Figure 2f,g). Just after binding, the rod-shaped bacteria tend to align their major axis approximately with the direction of the flow. With time, more bacteria attain a position approximately normal to the flow direction (Figure 2f). Bacteria attached with a large angle relative to the flow also tend to wiggle less than those aligned with the flow, as shown by the standard deviation of the binding angle. The realignment indicates that additional bonds, distributed along the bacterial rod, form between a bacterium and the surface. These bonds are strong enough to withstand the flow force that causes bacteria to align with flow. Analyzing the amplitude of the small motions around the median major axis of the bacterium can indicate the location and character of the attachment points under its body (Figure 2g).^74^ The WT *E. coli* binding to mannose-coated surfaces appear to rock slowly, rather than twist, symmetrically around their centers [Figure 2g (upper panel) and Supporting Movie 1]. The central part of the bacteria moves slightly less than the poles, the difference becoming even smaller toward the end of the experiment (Figure 2g, lower panel). We suggest that the realignment of the bacteria away from the flow direction and the symmetric rocking motion are characteristics of fimbriae-mediated binding to the surface since fimbriae are relatively long and distributed evenly over the bacterial body. This explains why binding is both flexible and homogeneous along the bacterial rod. Likely, the adhesion pattern changes with time due to increased fimbriae expression during the phase of adaptation to biofilm growth after binding. This interpretation is supported by a similar analysis made on *E. coli* that overexpresses fimbriae (Figure S3). For the heavily fimbriated phenotype, the initial adhesion pattern resembles the final pattern observed for WT *E. coli* in Figure 2f,g, and less change occurred over time.

### Bacterial Response to AMPs Provided in the Growth Media

**Figure 3:**
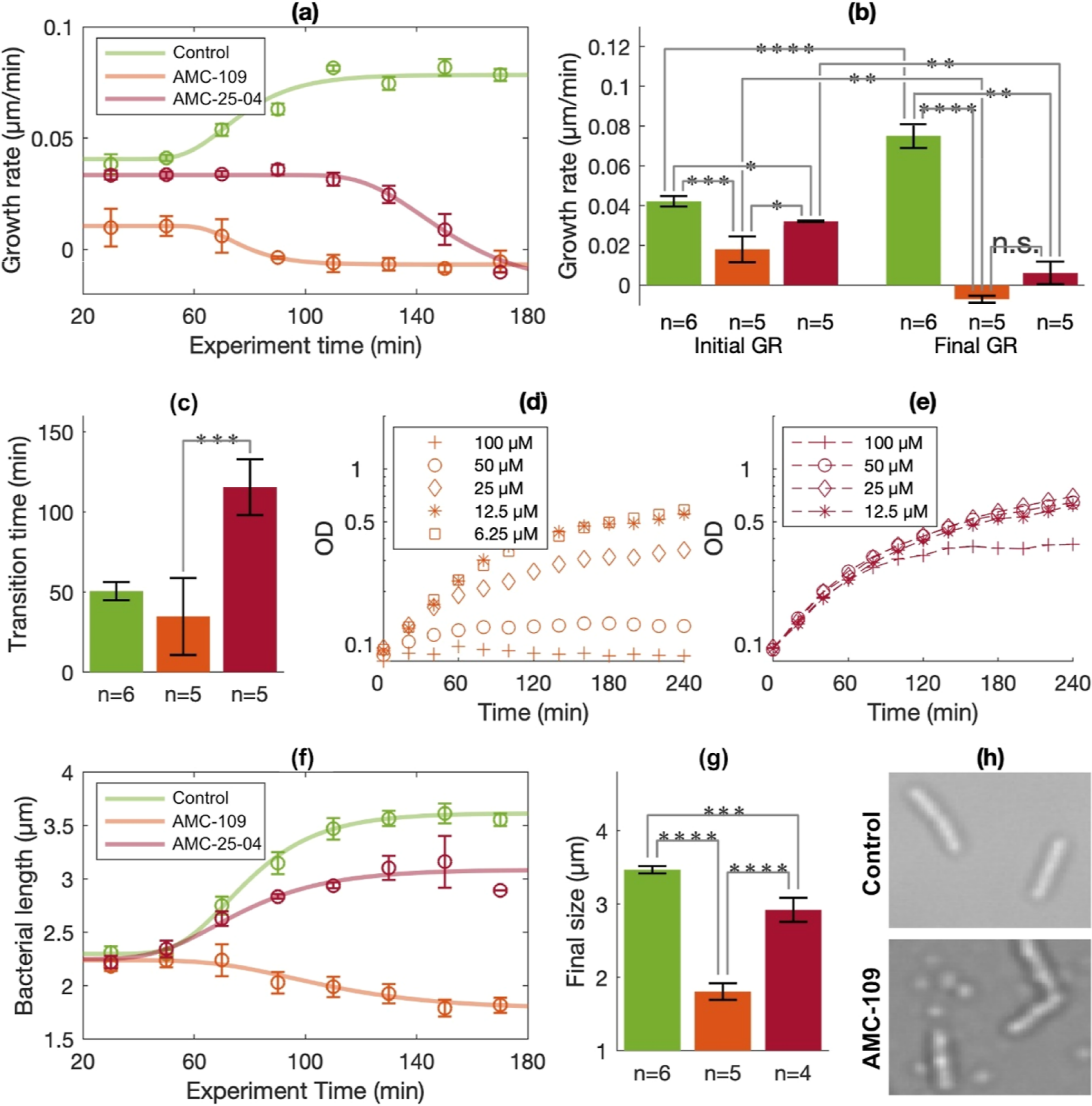
Efficacy of AMPs delivered in growth media. In all plots, green denotes control experiments (*N* = 6), orange denotes experiments with added AMC-109 (*N* = 5), and red denotes experiments with added AMC-25-04 (*N* = 5). Statistical significance was tested by Student’s *t*-test where n.s. denotes not significant, *p < 0.05, **p < 0.01, ***p < 0.005, and ****p < 0.001. (a) The plot shows the average and standard error (SE) of the median GR values for each bin in the boxplots (cf. Figure 2d) of all individual experiments. (b) The bars show the average and SE of median GRs measured before (“initial GR”) and after (“final GR”) the transition time was reached in the individual experiment. (c) Bars show the average and SE of the transition times determined in each experiment. The transition times correspond to the inflection points of the Gompertz’s fits to the data in the boxplots (cf. Figure 2d). (d) The plot shows batch-culture growth curves for *E. coli* in the presence of different concentrations of AMC-109. (e) The plot shows batch-culture growth curves for *E. coli* in the presence of different concentrations of AMC-25-04. (f) The plot shows the average and SE of the median bacterial size values for each bin in the boxplots (cf. Figure 2e) of all individual experiments. (g) Bars show the average and SE of median sizes measured after the transition time was reached in the individual experiment. (h) Micrographs detail the appearance of *E. coli* toward the end of a control experiment (upper panel) and an experiment where AMC-109 was added (lower panel). Both bacteria and AMP nanoparticles (small round features) are visible in the lower micrograph.

The normal growth of WT *E. coli* on mannose-coated surfaces was contrasted by a very different behavior observed when the growth media applied after binding was supplemented with 100 μM AMPs AMC-109 or AMC-25-04. The bacteria subjected to AMPs immediately showed attenuated GRs and, finally, growth arrest (Figure 3a). AMC-25-04 lowers the initial GR by 24% and AMC-109 by 57% relative to the control (Figure 3b).

The variation of the initial median GRs between replicates is small, particularly for the controls with pure growth media and experiments with medium supplemented with the less potent AMP AMC-25-04. This is notable since *E. coli* cultures had grown to reach slightly different OD in the LB media, and thus likely had different GRs, before they were injected to the flow system (cf. Materials and Methods section). The distribution of the initial GRs of the bacteria in a single experiment is also narrow (Figure S4). The GR attenuation observed on the population-level is accordingly not due to some bacteria being more sensitive to AMP and therefore reaching GR arrest early. It is thus clear that binding leads to a reset of the GR and that the degree of GR attenuation reflects only the surface-adhered state of the bacteria and the AMP-containing environment in combination. Interestingly, the same Cpx- and σ^E^-regulated envelop stress responses of *E. coli* are activated both in response to surface binding^34,38^ and as the bacteria’s first response to AMPs,^35,37^ suggesting a mechanistic link that may explain the apparent additive effect of surface binding and AMP treatment on the GR.

The time elapsed from the addition of AMPs to growth arrest is 116 min for AMC-25-04 and 35 min for AMC-109 (Figure 3c). However, as the median GR starts to decline, the distribution of the individual GRs also broadens, indicating that the transition to GR arrest is abrupt on the single-cell level and, in contrast to the GR attenuation, not synchronized for all cells. Taking either the initial GR attenuation or the typical time to GR arrest as measures of antibacterial efficacy, both observations indicate that AMC-109 is about three times more potent than AMC-25-04. This result compares well with data obtained from batch-culture experiments shown in Figure 3d,e. Particularly, growth curves measured for *E. coli* charged with 100 μM AMC-25-04 and 25 μM AMC-109 showed similar progression. The time to GR arrest is also similar in live-microscopy and batch-culture experiments. Yet, the initial attenuation of GR relative to the control observed for surface-bound bacteria subject to AMC-25-04 is not detectable in the corresponding batch culture experiments. Potentially, attenuation of GR due to the combined impact of surface binding and AMPs is more easily measured than the effect of AMPs alone, at least if the latter contribution is small. For the two AMPs tested in our experiments, the degree of initial GR attenuation correlated with the time needed to reach complete growth arrest, yet more AMPs must be analyzed to establish whether this relationship holds generally. Still, it is an intriguing possibility that the initial GR of surface-bound bacteria can be used as a measure of an AMP’s potency. The fact that bacteria show synchronized behavior in this early phase of biofilm formation provides a stable baseline for measuring already small deviations of the GR, as demonstrated above. Furthermore, the initial GR can also be measured relatively fast, and reliable values can be acquired within 1 h. Finally, the GR after surface binding is also a particularly relevant measure if the final aim is to establish AMPs for the purpose of antibacterial coatings since it directly reflects the biofilm formation process.

The progression of cell length was strongly impacted by AMPs (Figure 3f,g). Most strikingly, AMC-109 makes the cells shrink, which is partly due to bacteria dividing asymmetrically, creating small cells, and partly due to an actual, abrupt cell shrinkage taking place as bacteria die (Supporting Movie 2). These processes also lend an irregular shape to bacteria, characterized by an uneven intensity distribution and less well-defined edges than those of normally growing *E. coli* (Figure 3h). The effect of AMC-25-04 was initially less drastic, causing the bacteria to grow longer, although not as long as those in the controls without AMPs. Decreased cell length homeostasis is indeed a characteristic of *E. coli* growing while subject to stress.^70^ Toward the end of the experiment, a similar behavior was observed for bacteria subjected to AMC-25-04 as was seen for those bacteria subjected to AMC-109. A surprising observation was the formation of AMP nanoparticles, appearing on the surfaces early in experiments with AMC-109 and later AMC-25-04 (Figure 3h, lower panel). These particles were also present in water-based solutions of AMPs not containing bacteria (Figure S5), excluding the possibility that they are debris of dead cells. It was recently reported that AMC-109 can form 5 nm particle structures that potentiates its antibacterial action.^23^ The particles observed here are much larger (hundreds of nanometers in diameter), and it is unclear whether they impact the efficacy of the AMPs.

### Bacterial Response to AMP-Coated Surfaces

**Figure 4:**
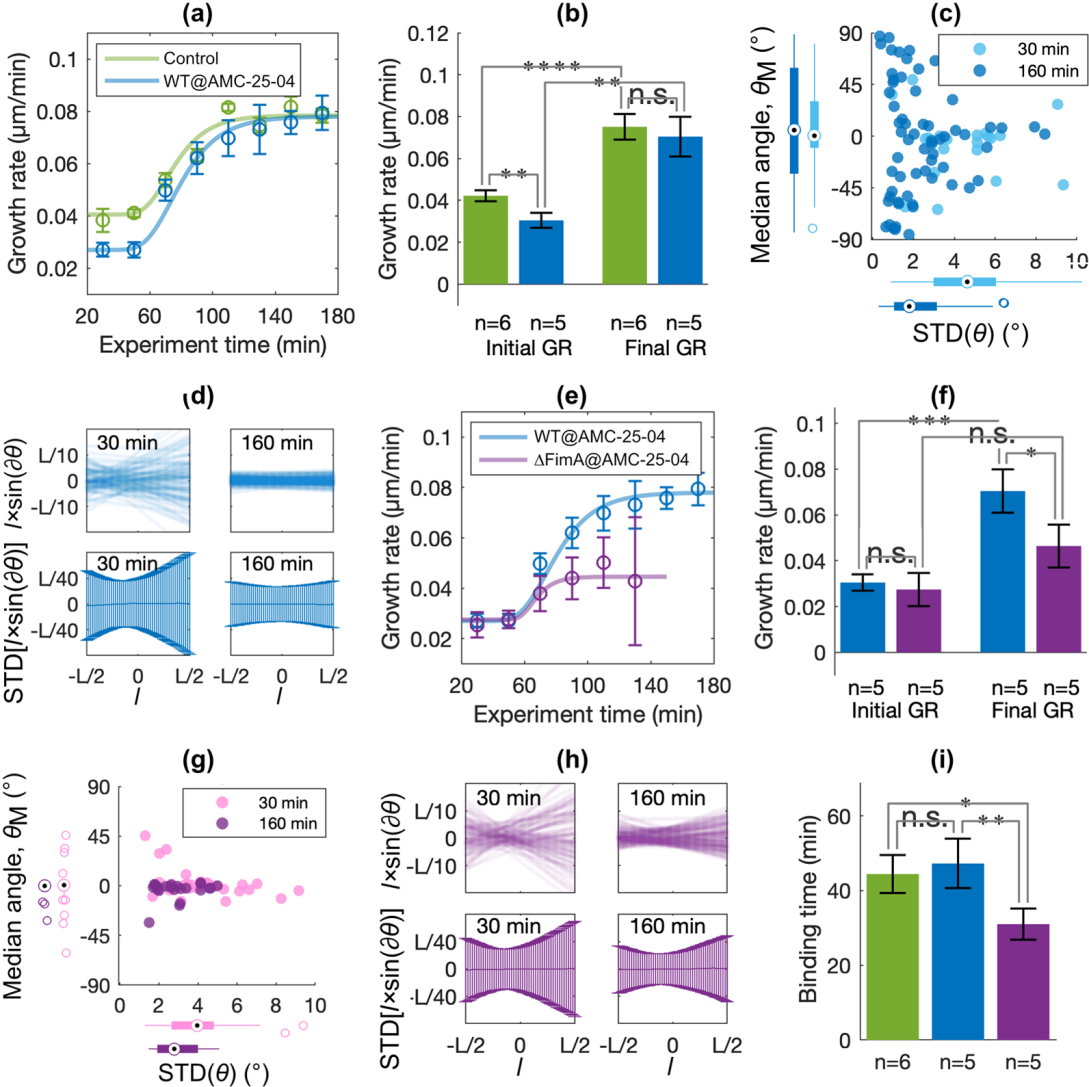
Efficacy of AMP-coated surfaces. In all plots, green denotes control experiments (*N* = 6), blue denotes experiments with WT *E. coli* (*N* = 5), and violet denotes experiments with fimbriae-deficient *E. coli*-Δ*FimA* (*N* = 5). Statistical significance was tested by Student’s *t*-test where n.s. denotes not significant, *p < 0.05, **p < 0.01, ***p < 0.005, and ****p < 0.001. (a) The plot shows the average and SE of the median GR values for each bin in the boxplots (cf. Figure 2d) of all individual experiments. (b) The bars show the average and SE of median GRs measured before (“Initial GR”) and after (“Final GR”) the transition time was reached in the individual experiment. (c) The combined scatter and boxplot show the median angle, *θ*_M_, versus the STD of *θ* for all bacteria at an early (30 min, light blue) and a late (160 min, dark blue) time point of the experiment (cf. Figure 2f). (d) Scatter plots in the upper panels show for a single example bacterium the instantaneous separations, *l* × sin(∂*θ*), between each position *l* along the bacterium’s major axis with total length *L* and the median major axis *M*. The bar plots in the lower panels show the distribution (standard deviations) of the instantaneous separations, *l* × sin(∂*θ*), for all positions *l* of all bacteria (c.f. Figure 2g). (e) The plot shows the average and SE of the median GR values for each bin in the boxplots (cf. Figure 2d) of all individual experiments. (f) The bars show the average and SE of median GRs measured before (“Initial GR”) and after (“Final GR”) the transition time was reached in the individual experiment. (g) The combined scatter and boxplot show the median angle, *θ*_M_, versus the STD of *θ* for all bacteria at an early (30 min, pink) and a late (160 min, violet) time point of the experiment (cf. Figure 2f). (h) Scatter plots in the upper panels show for a single example bacterium the instantaneous separations, *l* × sin(∂*θ*), between each position *l* along the bacterium’s major axis *L* and the median major axis *M*. The bar plots in the lower panels show the distribution (standard deviations) of the instantaneous separations, *l* × sin(∂*θ*), for all positions *l* of all bacteria (cf. Figure 2g). (i) Bars show the average and SE of bacteria’s mean binding times, *T*_1/2_, determined in the different experiments.

The WT *E. coli* bound readily to surfaces coated with AMC-25-04. The first 40–50 min after binding, the GR is attenuated by 26% relative to the GR of *E. coli* growing on mannose-coated surfaces (Figure 4a,b). During this period, bacteria also adhered differently than they did onto the mannose-coated control surfaces; in particular, they wiggled heavily (Figure 4c, light blue data). The wiggling can be best described as a twisting motion around a fixed point located somewhat upstream of the bacteria’s center points [Figure 4d (left panels) and Supporting Movie 3]. This pattern of movement is characteristic of an *E. coli* bacterium adhering to a surface via a single patch located on its body.^74^ Notably, while this contact patch is only a small fraction of the outer membrane, the overall impact of the grafted AMC-25-04 on the GR (−26%) is the same as when this AMP was supplied in the bulk media (−24%, cf. Figure 3a,b) where the entire membrane is targeted. The uptake of short AMPs from solution is typically rate limited by the lipid composition of the bacteria’s membrane, the negative net charges of the lipids, and the positive of the AMPs, respectively, giving rise to a force that pulls the AMPs into the membrane core. The number of AMPs that can be taken up spontaneously into the membrane thus saturates as the charge difference is equalized.^23^ Furthermore, most short AMPs are more easily taken up by Gram-positive (G^+^) bacteria rather than Gram-negative (G^–^) bacteria, e.g., *E. coli*, since the lipopolysaccharides (LPS) provide G^–^ bacteria with an additional outer barrier that withholds the AMPs from approaching the membrane core.^75^ Live-microscopy experiments have shown that while the surface of *E. coli* is completely covered by AMPs immediately after immersion in AMP solution, the uptake into the outer membrane can take significant time.^45^ These kinetic barriers, which limit the impact of AMPs in solution, are potentially less important for the uptake of covalently attached AMPs; in this case, the number of peptides protruding into the membrane will depend on the grafting density, the alignment of the AMP relative to the membrane, the geometry of the interface, and all the adhesion forces that pull the bacterial membrane and surface together. The latter is not limited to electrostatic attraction between individual AMPs and lipids but also includes colloidal forces between the bacterium as a whole and the surface^76^ as well as forces that are caused by the liquid flow. The fact that grafted AMC-25-04 inhibit bacterial growth to the same extent as AMC-25-04 delivered in bulk, although in the former case the total contact area is much smaller, implies that the concentration of AMPs in the contact patch is much higher than what is possible to achieve due to passive uptake and/or that these AMPs are more persistent, thus lending a higher antibacterial efficacy per molecule. From a materials design perspective, this finding is important since it shows that an antibacterial coating can function even if the contact area between the bacterial body and the material is small.

After approximately 1 h, the*E. coli* bound to the AMP-coated surfaces changed behavior drastically: The previously attenuated GR increases in a similar way as seen for the mannose-coated controls, and after 100 min, the GRs are the same (Figure 4a,b). The change of GRs was timely synchronized, implicating that the vanishing effect of the AMP coating is caused by a bacterial adaptation for biofilm growth. Also, the bacteria’s binding mode shifted to resemble that of the *E. coli* growing on mannose-coated surfaces, characterized by realignment away from the flow direction and less wiggling (Figure 4c, dark blue data), the latter involving a symmetric rocking motion around the bacteria’s centers [Figure 4d (right panels) and Supporting Movie 4]. We suggest that the diminishing sensitivity to the AMP coating may relate to this transition from surface adhesion via a patch of the bacterial membrane to a mode where the bacteria bind to several distributed contact points through fimbriae, the expression of which increases during biofilm adaptation. Notably, fimbriae are numerous, micron-long, and comparably stiff structures that will push a bacterium slightly away from the surface.^77^ This lowers the chance that the bacterial outer membrane contacts the coating, which extends just a few nanometers. To test the hypothesis, we studied the surface growth of a mutant, *E. coli*-Δ*FimA*, that can produce the same proteins as WT *E. coli* but for FimA, the major structural protein of the fimbrial rod, making these bacteria nonfimbriated (Figure 4e,f). *E. coli*-Δ*FimA* adhere to the AMP coating at about the same rate as WT *E. coli* and initially grow at the same, reduced rate. However, in contrast to WT *E. coli*, the GR does not increase to the same extent after the lag-phase for the bacteria without type-1 fimbriae: the final GR remained significantly lower (−34%) than that measured for WT *E. coli*. In the absence of type 1 fimbriae, *E. coli*-Δ*FimA* remained aligned with the flow direction (Figure 4g) and displayed an asymmetric twisting motion [Figure 4h (left panels) and Supporting Movie 5], indicating that binding via the initial adhesion patch prevailed throughout the experiment, although some stiffening of the bond could still be observed [Figure 4h (right panels) and Supporting Movie 6].

Planktonic wild-type *E. coli* bacteria are known to have low fimbriae expression as opposed to those growing in biofilms on tissue or catheters in the urinary tract, which are heavily fimbriated.^78,79^ This explains our observation that both wild-type *E. coli* and the nonfimbriated mutant have the same adhesivity and directly after binding form a similar contact patch with the coating. The fact that both strains initially behave similarly also excludes the possibility that the differences observed 90 min after binding are due to solvated fimbriae proteins present in the bacterial solution that adsorb to and cover the AMPs during the first 20 min of the experiment. Furthermore, type 1 fimbriae are extraordinarily stable structures that do not disintegrate easily^80^ and detached intact fimbriae are only sparsely present in solution even when the fimbriae expression is elevated (Figure S6). Notably, although *E. coli*-Δ*FimA* bound readily to the AMP-coated surface, the daughter cells often left the surface shortly after division. This, in combination with that the initially bound bacteria occasionally left too (as is also observed for WT *E. coli*), results in a characteristic binding time of 27 min, which is less than half of that seen for WT *E. coli* (Figure 4g). The total number of surface-bound bacteria consequently decreases over time, and after 140 min, reliable analysis of GR was not possible anymore (Figure 4i). We believe that the difference between the strains with respect to the release rate of daughter cells reflects the functional mechanism of type-1 fimbriae that modulates the colonization of surfaces under flow.^79^ The different outcomes of cell division for fimbriated and nonfimbriated bacteria, although the adhesiveness of the coatings is the same, is an additional example of how phenotypical changes during biofilm formation may impact bacterial behavior stronger than the surface properties, in this case its hydrophobicity and positive charge.

With respect to their ability to form biofilms, the WT *E. coli* bacteria investigated here are seemingly less inhibited by a covalent coating of small AMPs than are *Staphylococcus epidermidis* bacteria, which were investigated in a previous study employing the CERTIKA method for the evaluation of antibacterial efficacy.^29^ It can be tempting to attribute this difference to the generally low intrinsic activity of short AMPs against G^–^ bacteria caused by the presence of LPS. However, our results obtained for WT *E. coli*, in the early phase of the experiment, and for nonfimbriated *E. coli*-Δ*FimA* show that the grafted AMPs in the small contact patch that define the interface between the coating and the bacteria in fact have a higher intrinsic antibacterial potency than the same AMP provided in solution. The relatively low, overall antibiofilm activity of the coating can instead be attributed to the bacteria’s ability to escape direct contact between the outer membrane and the surface by increased expression of fimbriae after binding. Type-1 fimbriae are the most common type of pili on *Escherichia*, but it is also present on other G^–^ bacteria, e.g., *Klebsiella* and *Pseudomonas*, implicated in biofilm formation on biomaterials. Many other types of pili found on G^–^ have similar appearance and mechanical properties^81^ and may thus give rise to the same effect. Gram-positive bacteria, however, produce pileous extensions that are structurally and mechanically different;^82^ thus, it remains to be investigated whether G^+^ bacteria, for example *Staphylococcus*, can moderate surface adhesion postbinding in a way that lowers their sensitivity to an AMP coating. Our work expands the current understanding about factors that influence the efficacy of AMP-based antimicrobial coatings: While it has been previously established that amino acid sequence,^28,29^ the tether length/position,^28,29,58^ and the chemistry of the support^57,60^ all have a strong impact, we here show that the nature of the physical contacts that bacteria form with surfaces can be equally important. Many phenotypical changes of bacteria, which include but are not limited to their attachment, occur during early biofilm formation in response to surface binding.^38^ Identifying the features that govern different bacteria’s surface adaptation process and developing materials that can mitigate this are thus important tasks for future research in the area.

## Conclusions

The combination of live-microscopy and microfluidics used in this work is superior to classical batch-culture methods in measuring the efficacy of antibacterial surface coatings: Under settings that reasonably well mimic the intended final usage, we could distinguish and quantify the contribution of several different antibacterial mechanisms that with other methods appear to be intertwined and not possible to separate. Particularly, our proof-of-concept study featuring *E. coli* bacteria identified the possibility that the rate of bacterial elongation right after binding to a surface reflects the intrinsic antibacterial efficacy of both AMPs in solution and AMP coatings. This measurement is appealing for ranking of the performance of different AMPs since already small effects can be detected in a relatively short measurement. Furthermore, our implementation features the simplest possible microscopy, automated analysis, and generally applicable click-chemistry for surface modification, making the platform suitable for scaling-up and medium-throughput analysis. The analysis also allowed us to separate the impact of the AMP’s intrinsic molecular antibacterial potential from the impact of events relating to the biological complexity of biofilm formation on the overall efficacy of the AMP coating. Although the intrinsic antimicrobial potency of the grafted AMPs was higher than that for the same AMP provided in solution, this effect could be exerted only on the planktonic phenotype of *E. coli*, which still dominates initially after binding, but not on the phenotype evolving during the postbinding lag-phase. The difference relates to a transition from an early adhesion mode involving a patch of the bacterial membrane being in contact with the coating to a mode where the bacteria bind to several distributed contact points via type-1 fimbriae, a type of extension frequently occurring on the body of *E. coli* and other several biofilm-forming Gram-negative bacteria. These findings have important implications for the design of covalent antibacterial coatings with activity toward Gram-negative bacteria: It shows that already a minor contact between the bacterial membrane and the surface coating can be sufficient to get an antibacterial effect. Yet, to sustain this effect, the coating must intervene with the bacteria after binding in such a way that the progression of the biofilm-adapted, more heavily fimbriated phenotype is halted. Alternatively, strategies for covalent tethering must be used that can respond to the bacteria’s shift in binding mode, maintaining the direct contact between the grafted AMPs and the bacterial membrane. A more detailed knowledge about how different types of bacteria change from planktonic to biofilm growth may ultimately enable the construction of coating with selective antibacterial properties.

## Supporting Information Available

^1^H NMR spectrum of AMC-25-04 in DMSO-*d*_6_, calculated flow profiles in the microfluidic channel, bacterial alignment and small movement for *E. coli*-*Fim*+, GRs for *WT**E. coli* in LB + 100 μM AMC-25-04, nanoparticle tracking analysis of AMP particle formation in solution, and TEM micrograph of LB media after growth of fimbriated *E. coli* (PDF)
Small motions of *WT**E. coli* bound to mannose-modified surface (25 times real speed) (AVI)
Shrinking bacterium (250 times real speed) (AVI)
Small motions of *WT**E. coli* bound to AMP-modified surface at 30 min (25 times real speed) (AVI)
Small motions of *WT**E. coli* bound to AMP-modified surface at 160 min (25 times real speed) (AVI)
Small motions of *E. coli*-Δ*FimA* bound to AMP-modified surface at 30 min (25 times real speed) (AVI)
Small motions of *E. coli*-Δ*FimA* bound to AMP-modified surface at 160 min (25 times real speed) (AVI)

## Supplementary Material

am3c16004_si_001.pdf

am3c16004_si_002.avi

am3c16004_si_003.avi

am3c16004_si_004.avi

am3c16004_si_005.avi

am3c16004_si_006.avi

am3c16004_si_007.avi
