# Supplementary material for: Preventing E. coli Biofilm Formation with Antimicrobial Peptide-Functionalized Surface Coatings: Recognizing the Dependence on the Bacterial Binding Mode Using Live-Cell Microscopy
Source: ACS Appl Mater Interfaces. 2024 Jan 31;16(6):6799–812. doi: 10.1021/acsami.3c16004 (PMC10875647; doi:10.1021/acsami.3c16004)
Supplement: Supplementary file 1 — am3c16004_si_001.pdf [file am3c16004_si_001.pdf]

Supporting Information

Preventing *E. coli* Biofilm Formation with Antimicrobial Peptide-Functionalized Surface Coatings: Recognizing the Dependence on the Bacterial Binding Mode Using Live-Cell Microscopy

Adam Hansson[1,4], Eskil André Karlsen[2,3], Wenche Stensen[3], John S. M. Svendsen[2,3], Mattias Berglin[1,4] and Anders Lundgren[1,5*]

[1]Department of Chemistry and Molecular Biology, University of Gothenburg, Gothenburg, 40530, Sweden

[2]Amicoat A/S, Sykehusvegen 23, Tromsø, 9019, Norway

[3] Department of Chemistry, UiT The Arctic University of Norway, Tromsø, 9037, Norway

[4]Department of Chemistry and Materials, RISE Research Institutes of Sweden, Borås, 50115, Sweden.

[5]Centre for Antibiotic Resistance Research (CARe), University of Gothenburg, Gothenburg, 41346, Sweden

[*]To whom correspondence should be addressed: Anders Lundgren, phone: +46 709692031, e mail: anders.lundgren@gu.se, ORCID: 0000-0002-8537-9974

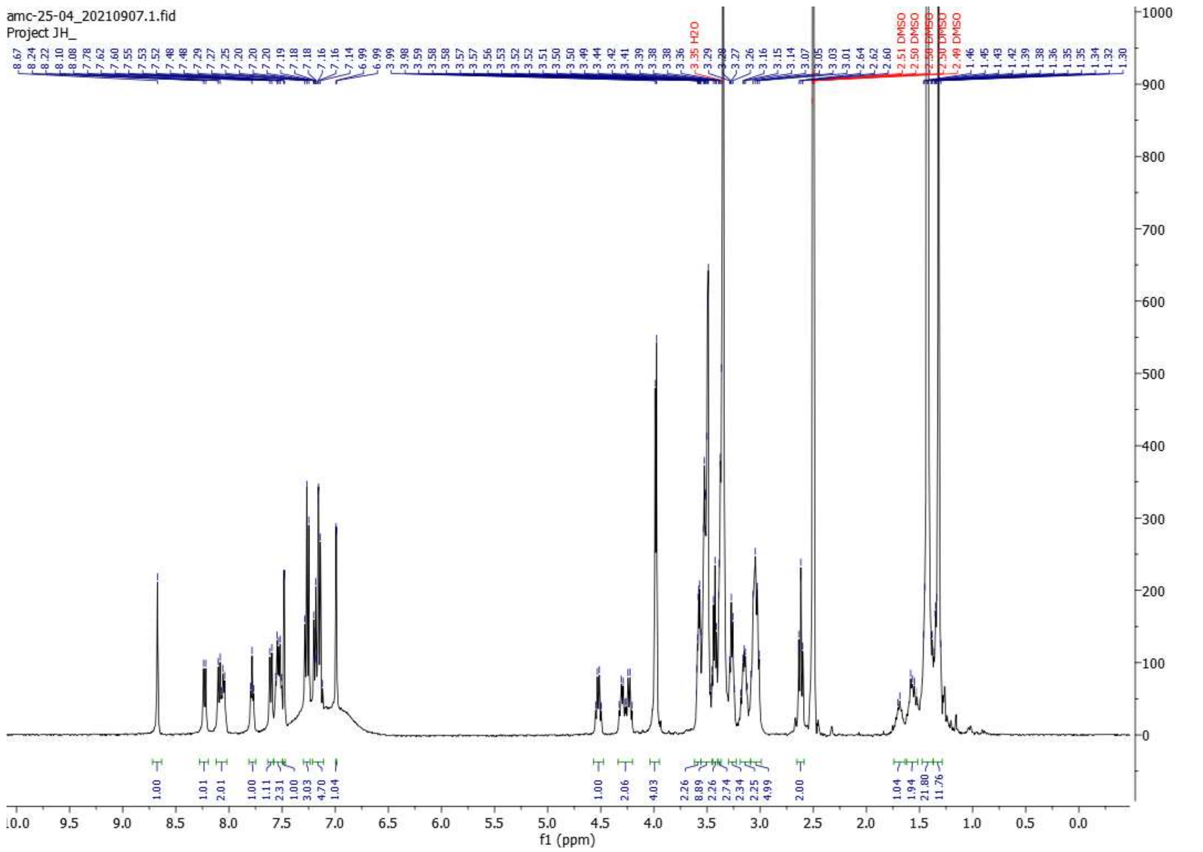

Supporting Figure S1: ^{1}H NMR spectrum of AMC-25-04 in DMSO-*d6*.

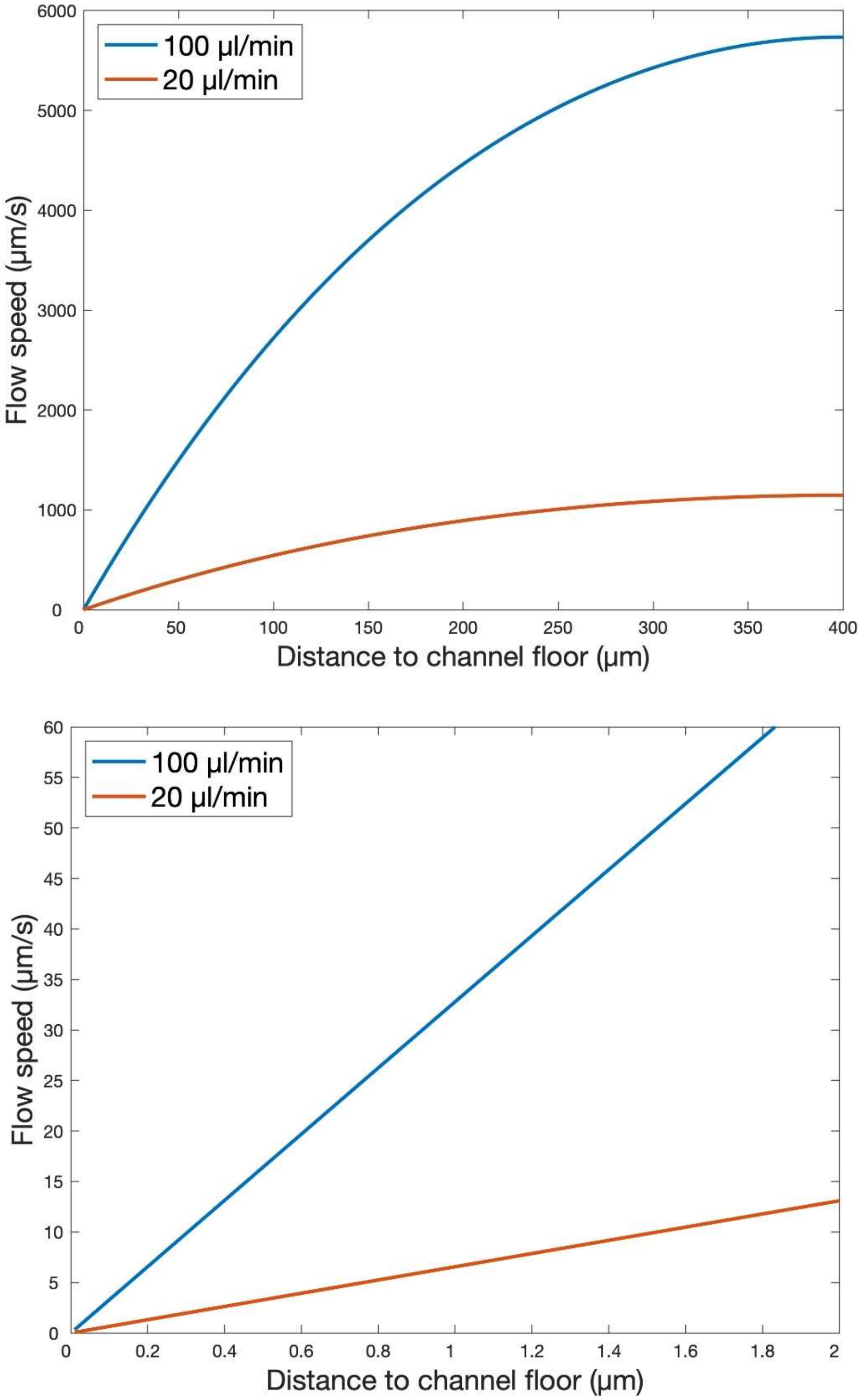

Supporting Figure S2: Calculated flow profiles showing the flow speed profile in the lower half of the channel (upper panel) and close to the bottom of the channel where the bacteria stick (lower panel).

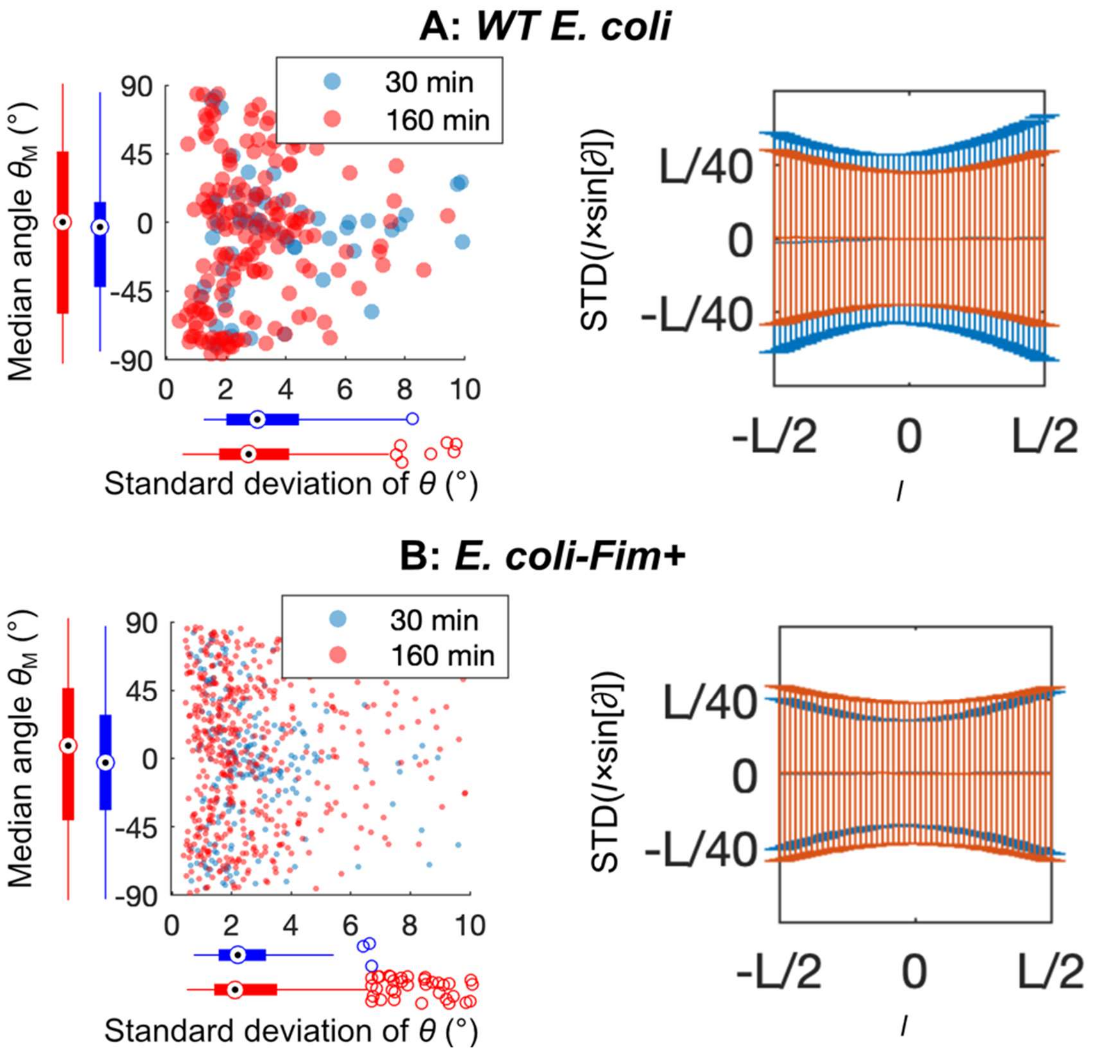

Supporting Figure S3: The combined scatter and boxplots show the median angle, θ_M, versus the standard deviation (STD) of θ for all bacteria at an early (30 min, blue points) and a late (160 min, red points) time point of the experiment. The bar plots to the right show the distribution (standard deviations) of the instantaneous separations, l×sin(∂θ), for all positions l of all bacteria present early (30 min, blue bars) and late (160 min, red bars) in the experiment. **(A)** *WT E. coli* **(B)** *E. coli-Fim+*.

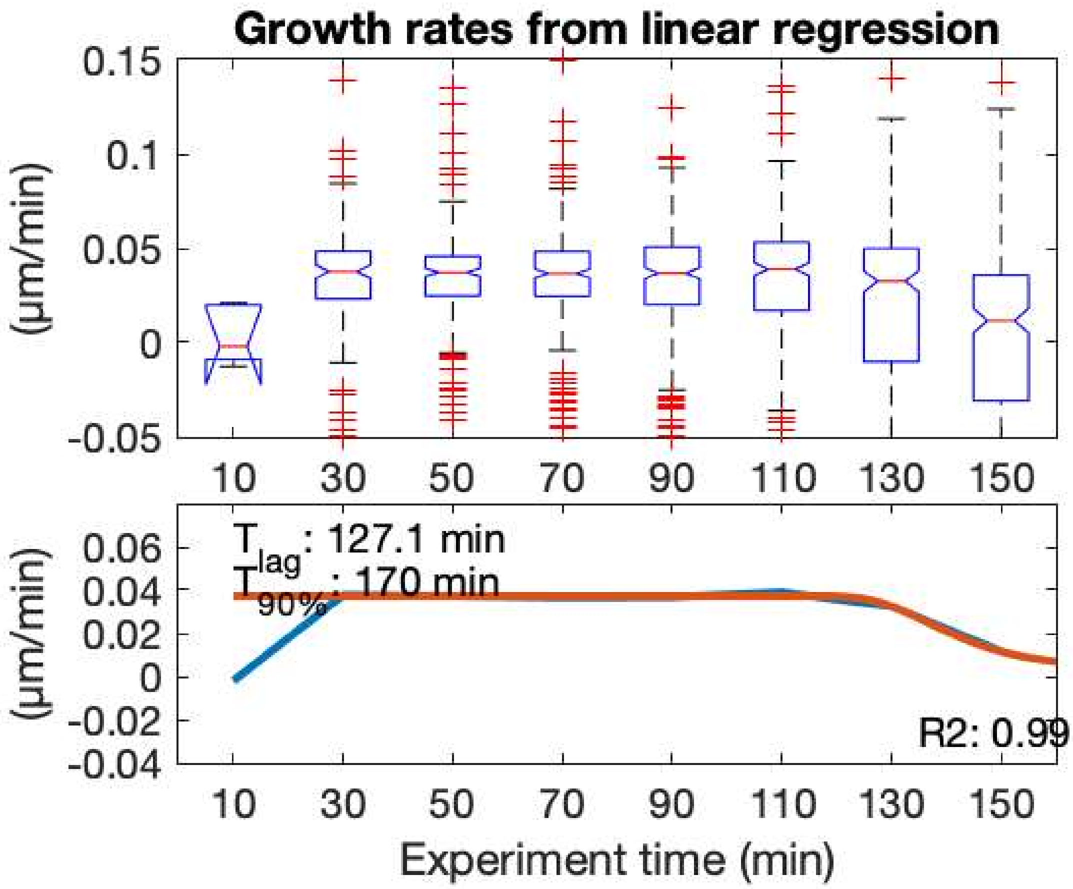

Supporting Figure S4: Boxplot showing the distribution of GRs of the surface-bound bacteria charged with 100 µM AMC-25-04 in the LB growth media. Data for t<20 min. i.e. during the injection phase, is not representative since few bacteria could be traced >10 min during the binding phase. Note that the distribution of GRs is narrow in the beginning of the experiment but broadens towards the end.

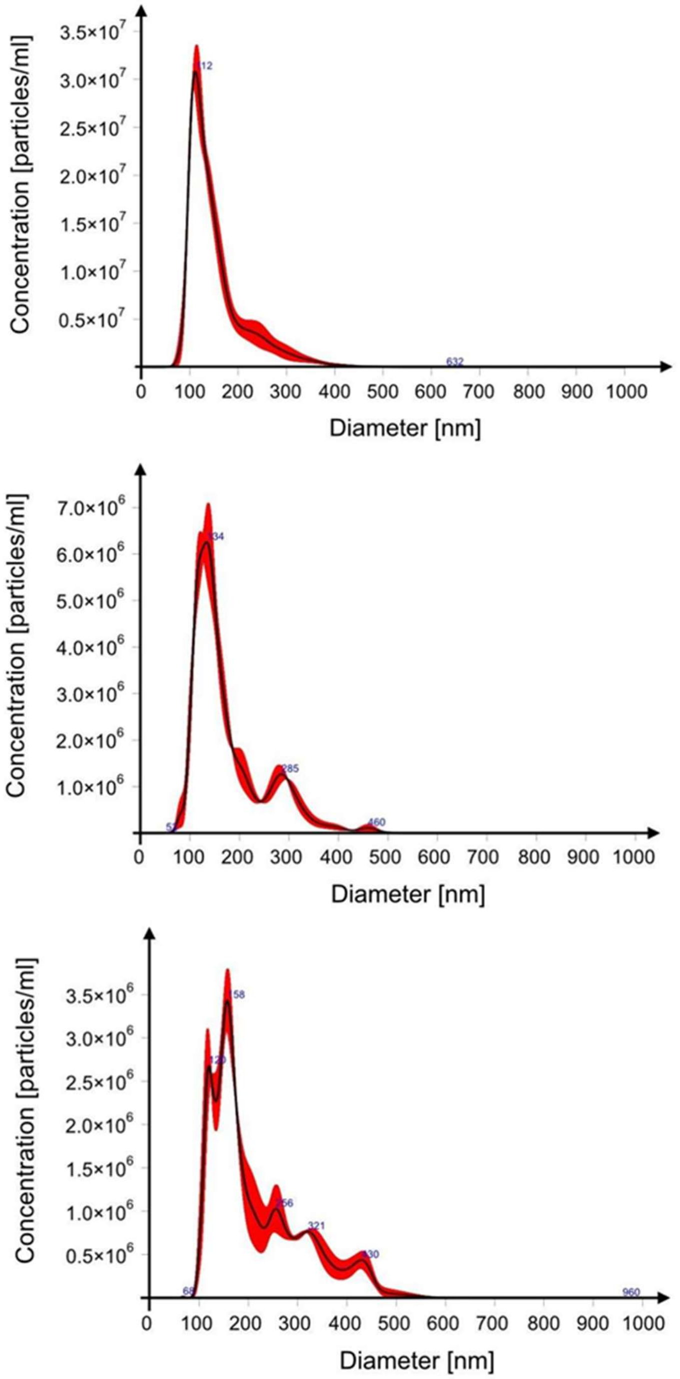

Supporting Figure S5: Nanoparticle tracking analysis (NTA) measurements showing the concentration and size distribution of AMP nanoparticles obtained when dissolving AMC-109 to a concentration of 100 µM in TRIS-buffer. The solution was drawn through the fluidic system at a flow rate of 10 µL/min and at each time point data were acquired for 3 times 60 second. The graphs show the concentration and size distribution of the particles (the three captures were merged) 5 minutes (top), 60 minutes (middle), 180 minutes (bottom) after dissolving the peptide. The initially formed particles with size around 100 nm appear to aggregate over time and form larger particles of various sizes up to 500 nm. Our preliminary analysis shows that the particulate fraction of the AMPs remains constant over time. Assuming that the first formed smaller particles are homogenous and filled by the AMPs we estimate that the particulate fraction is less than 10% of the total AMP concentration.

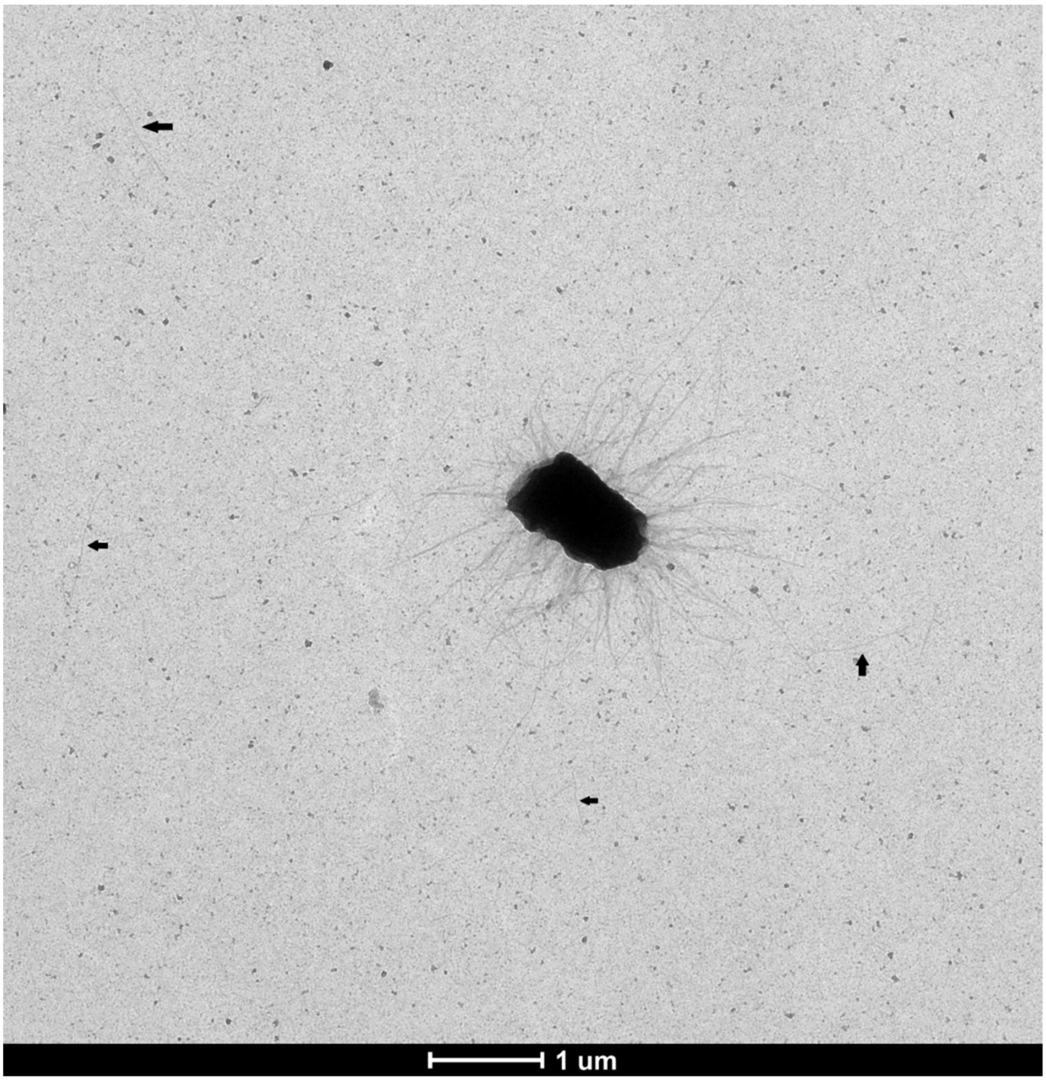

Supporting Figure S6: TEM micrograph showing an *E. coli* bacterium that overexpress type 1 fimbriae (the K12 strain PC31 Δ*fim* Km^r harboring the plasmid pPKL4 that encode all *fim* genes[1]) deposited directly on the TEM grid from an overnight culture made in LB media. EM grids coated with a 3–4 nm thick carbon film (CF300-CU-UL, Electron Microscopy Sciences, USA) were treated in an UV/ozone chamber (ProCleaner, Bioforce Nanoscience, USA) for 1 minute and then put up-side-down on a droplet of bacterial solution for 1 minute to allow bacteria to adsorb. The adsorbed material was fixed with glutaraldehyde (2.5% w/w solution in PBS buffer pH 7.4) for 10 minutes, rinsed 3×10 seconds with MQ water and then negatively stained with uranylacetate (1% w/w solution in MilliQ water) for 1 minute. Electron micrographs were captured using a FEI Tecnai G2 microscope operated at 160 kV acceleration voltage. A few identified detached Type 1 fimbriae are indicated by the black arrows.

(1) Klemm, P.; Jrgensen, B. J.; van Die, I.; de Ree, H. The Fim Genes Responsible for Synthesis of Type 1 Fimbriae in Escherichia Coli, Cloning and Genetic Organization. *Mol. Genet. Genomics* **1985**, *199*, 410–414. https://doi.org/10.1007/bf00330751.
